# Treatment outcomes in recurrent versus *de novo* metastatic pancreatic adenocarcinoma: a real world study

**DOI:** 10.1186/s12885-022-10130-4

**Published:** 2022-10-12

**Authors:** Laura Miotke, Christopher Nevala-Plagemann, Jian Ying, Vaia Florou, Benjamin Haaland, Ignacio Garrido-Laguna

**Affiliations:** 1 Division of Medical Oncology, Huntsman Cancer Institute, 2000 Circle of Hope, Salt Lake City, UT 84112 USA; 2 Department of Internal Medicine, University of Utah School of Medicine, 30 North 1900 East, Salt Lake City, UT 84132 USA; 3 Department of Population Health Sciences, 295 Chipeta Way, Salt Lake City, UT 84108 USA

**Keywords:** Real-world outcomes, Overall survival, Pancreatic ductal adenocarcinoma, Recurrent, De Novo

## Abstract

**Background:**

A majority of patients undergoing curative intent surgery for pancreatic ductal adenocarcinoma (PDAC) will unfortunately develop recurrent disease. Treatment outcomes for patients with metastatic disease remain suboptimal. In this study, we evaluated clinical outcomes of patients with recurrent PDAC who received systemic therapy and compared outcomes to patients with *de novo* metastatic PDAC undergoing systemic therapy.

**Methods:**

Patients diagnosed with metastatic PDAC between 2014 and 2019 were included using a real-world database. Patients were characterized as either *de novo* or recurrent based on the date of metastatic diagnosis and history of surgical resection. Overall survival (OS) was summarized within groups via Kaplan–Meier survival estimates and compared using Cox proportional hazards models.

**Results:**

We included 5170 patients with metastatic PDAC, of which 1101 (21.3%) were classified as having recurrent disease. Median OS for the recurrent group was significantly greater at 10.8 m (95% CI 9.9–11.7) than in the *de novo* group at 7.3 m (95% CI 7.0–7.7, *p* < 0.001). We did not observe a significant difference in OS based on when patients recurred after surgery: 10.0 m (95% CI 8.7–11) within six months of surgery versus 11.6 m (95% CI 10–12, *p* = 0.256) greater than six months from surgery.

**Conclusions:**

These data support the inclusion of patients with recurrent PDAC in clinical trials for advanced disease, including those who develop recurrent disease within six months of surgery. Due to observed differences in survival, randomization should be stratified by disease presentation (recurrent vs *de novo*).

## Background

Pancreatic cancer is currently the fourth leading cause of cancer-related deaths in the U.S. It is estimated that it will account for 62,210 new cases in 2022 and may become the second leading cause of cancer related death by 2040 [1]. By the time PDAC is diagnosed, patients often have advanced, incurable or rapidly progressive disease driving these poor outcomes [2]. Median overall survival for these patients remains at just over a year according to MPACT and PRODIGE-4 studies [3, 4].

For the minority of patients that present with early-stage disease, recent advances in neoadjuvant and adjuvant treatment strategies have improved outcomes [5–9]. Despite these advances, most patients with PDAC that undergo resection will eventually develop recurrent disease. For example, in the CONKO-001 trial, which at the time of publication established adjuvant gemcitabine as the standard of care, 87% of the 354 patients enrolled eventually relapsed [7]. Similarly, in the ESPAC-4 and PRODIGE-24 trials, which demonstrated superiority of multiagent cytotoxic regimens over adjuvant gemcitabine alone, the recurrence rates were 65% and 51%, respectively [5, 6].

One potential explanation for the high recurrence rates seen in adjuvant trials is the idea that, in almost all cases, PDAC is a systemic disease at the time of diagnosis regardless of appearance on imaging. This hypothesis is supported by the fact that in genetically engineered mouse models, PDAC cells have been shown to metastasize even before there was a detectable pancreatic tumor [10, 11]. In one of these works, the authors used a Cre-lox based mouse model of PDAC and a lineage-labelling system to track pancreatic epithelial cells during cancer progression. The authors showed that even at early stages of disease, where only premalignant pancreatic intraepitheliel neoplasia (PanIN) lesions could be seen on imaging, pancreatic cells had already seeded the liver of those mice [11]. In another study, the authors used diagnosis and tumor distribution data from PDAC patients with autopsies to create an exponential mathematical model that predicted the rates and patterns of pancreatic cancer growth and dissemination. This estimated that a majority of patients harbor metastasis at diagnosis [12]. In another study, taxonomic modelling of whole exome sequencing data from primary, recurrent and metastatic tumor samples indicated that metastasis has occurred prior to surgery [13]. Clinically, the PREOPANC study showed that a quarter of the patients randomized to the immediate surgery group did not undergo resection due to metastatic or locally advanced disease at the time of staging laparoscopy or laparotomy [14].

Given these findings, there has been increasing interest in neoadjuvant treatment strategies as a potential way to gain early control of micrometastatic disease [8, 14–17]. However, long terms outcomes remain poor, even for patients with resectable disease. Thus, for a majority of PDAC patients who undergo resection, the goal of treatment will at some point shift from curative intent to a palliative approach. Over the last decade, there have also been practice-changing advances in managing locally advanced or metastatic PDAC. Most notably, the PRODIGE-4 and MPACT trials established FOLFIRINOX and Gemcitabine plus nab-paclitaxel as the standard-of-care first-line regimens in the metastatic setting, respectively [3, 4]. Whether the results of these landmark trials can be applied to patients with recurrent disease after resection is still largely unknown. Patients who received prior chemotherapy or radiation were excluded from the PRODIGE-4 trial and only a small percentage (7%) of patients in the MPACT trial had a previous Whipple procedure. Additional prospective data is clearly needed in order to better understand how to manage patients with recurrent disease.

One potential explanation for the exclusion of recurrent PDAC patients in clinical trials is that there is a relative paucity of data on the general outcomes of these patients. A recent single-center, retrospective study demonstrated improved overall survival in patients with recurrent PDAC who received multiagent chemotherapy regimens compared to those who received no therapy or a single agent [15]. Overall survival of patients in this study was similar to the reported survival of patients with *de novo* metastatic disease. To our knowledge, however, there have been no extensive, multicenter studies that have compared treatment outcomes of recurrent PDAC versus *de novo* metastatic PDAC in the United States. In this study, we utilize a large, well-validated database to describe the real-world outcomes of patients who received treatment for recurrent PDAC.

## Methods

### Data source

This study utilized de-identified electronic health record-derived data from the Flatiron Health database. This nationwide, longitudinal database is comprised of patient-level structured and unstructured data, curated via technology-enabled abstraction methods [18, 19]. Patients included in this dataset originate from both community and academic oncology settings. During the study period, the data originated from approximately 280 cancer clinics representing approximately 800 sites of care. Institutional Review Board approval of the study protocol was obtained before the study was conducted and included a waiver of informed consent.

### Study cohort

The Flatiron Health database was queried for patients diagnosed with metastatic PDAC, stage IV disease or earlier stage disease with subsequent recurrence or progression of disease, between January 2014 and October 2019. Patients who did not receive chemotherapy for metastatic disease were excluded from the analysis. To ensure the adequacy of treatment and outcome data, any patients without a visit or medication order within 90 days of metastatic diagnosis were excluded. Patients meeting the study criteria were classified as either *de novo* metastatic PDAC or recurrent PDAC based on record of surgery and date of metastatic disease diagnosis. Patients with recurrent PDAC were further stratified based on whether they developed recurrent disease greater than or less than six months after surgery.

### Statistical analysis

Differences in baseline characteristics of patients with *de novo* metastatic or recurrent PDAC were compared using a chi-square test for categorical variables and Wilcoxon test for continuous variables. Overall survival (OS), defined as the time from the date of metastatic diagnosis to the date of death, based on a composite real-world mortality endpoint, was reported within groups via the Kaplan–Meier method [20]. OS of the groups were compared with a log-rank test. Patients were censored based on their last documented entry in the electronic health record. To control for potential confounders, OS between groups was compared utilizing a Cox proportional hazards model adjusting for gender, age, race, ECOG performance status, smoking status, primary tumor site, baseline CA 19–9 within 30 days of metastatic diagnosis, albumin, lymphocyte, neutrophil and monocyte levels, and first-line palliative systemic therapy received.

## Results

### Study cohort characteristics

**Fig. 1:**
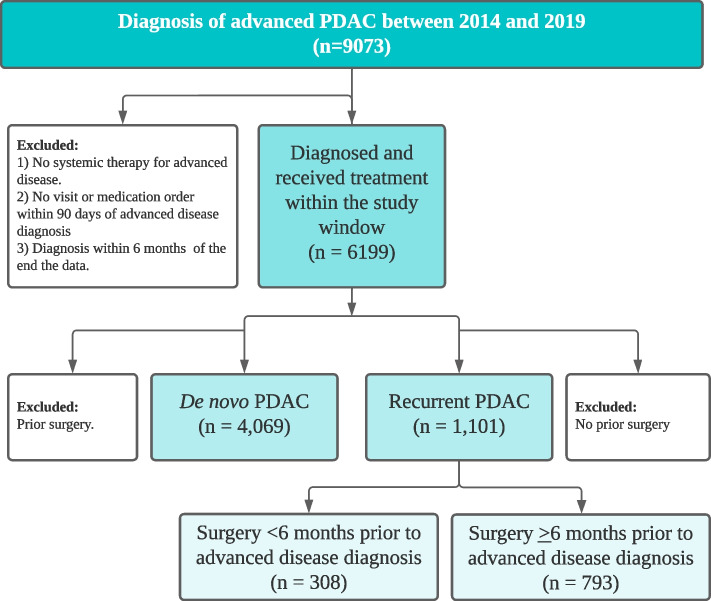
Study design. PDAC, pancreatic ductal adenocarcinoma

Out of the 9,073 patients with metastatic PDAC in the database, 5,170 patients met the criteria for inclusion in this study. Within this group, 21.3% (*n* = 1,101) met the criteria for having recurrent disease. The majority (73%) of patients with recurrent PDAC developed recurrent disease greater than six months after surgery (Fig. 1).

Statistically significant differences between baseline characteristics of patients in the recurrent and *de novo* PDAC groups were noted with regard to site of the primary tumor (71% head of the pancreas in the recurrent group compared to 40% in *de novo* group), median CA 19–9 at time of metastatic diagnosis (92.8 U/ml in the recurrent group vs. 617 U/ml in the *de novo* group) performance status (65% ECOG 0 to 1 in the recurrent group compared to 51% in the *de novo* group), and first line palliative therapy received. Patients with missing data were not excluded from analysis. Missing data was treated as a separate variable in each category which was then adjusted for in the multivariable cox regression analysis. Baseline characteristics are summarized in Table 1.

**Table 1:** Patient demographics and clinical characteristics

	**All** **(*n* = 5170)**	**De novo** **(*n* = 4069)**	**Recurrent** **(*n* = 1101)**	***P***-value
**Age at Diagnosis**	68 (61–75)	68 (61–75)	68 (61–74)	0.94
**Gender**				
**Male**	2838 (55%)	2248 (55%)	590 (54%)	0.33
**Female**	2332 (45%)	1821 (45%)	511 (46%)	
**Race**				
**Asian**	85 (2%)	69 (2%)	16 (1%)	< 0.001
**Black or African American**	433 (8%)	359 (9%)	74 (7%)	-
**White**	3593 (69%)	2769 (68%)	824 (75%)	-
**Other**	559 (11%)	435 (11%)	124 (11%)	-
**Not Specified**	500 (10%)	437 (11%)	63 (6%)	-
**ECOG at metastatic diagnosis**				
**0–1**	2779 (54%)	2061 (51%)	718 (65%)	< 0.001
**2 + **	603 (12%)	493 (12%)	110 (10%)	
**Unknown**	1788 (35%)	1515 (37%)	273 (25%)	
**Treatment Received**				
**Gemcitabine, nab-paclitaxel**	2471 (48%)	2018 (50%)	453 (41%)	< 0.001
**Fluoropyrimidine-based doublet**	304 (6%)	160 (4%)	144 (13%)	
**FOLFIRINOX**	1374 (27%)	1145 (28%)	229 (21%)	
**Single-agent cytotoxic chemotherapy**	639 (12%)	445 (11%)	194 (18%)	
**Non-standard Combination**	382 (7%)	301 (7%)	81 (7%)	
**Stage at diagnosis**				
**I**	106 (2%)	0 (0%)	106 (10%)	< 0.001
**II**	754 (15%)	0 (0%)	754 (68%)	
**III**	123 (2%)	0 (0%)	123 (11%)	
**IV**	4069 (79%)	4069 (100%)	0 (0%)	
**Unknown**	118 (2%)	0 (0%)	118 (11%)	
**Primary Site**				
**Body**	984 (19%)	878 (22%)	106 (10%)	< 0.001
**Head**	2402 (46%)	1619 (40%)	783 (71%)	-
**Tail**	1050 (20%)	913 (22%)	137 (12%)	-
**Overlapping sites**	526 (10%)	464 (11%)	62 (6%)	-
**Not specified**	208 (4%)	195 (5%)	13 (1%)	-
**Smoking Status**				
**Smoker**	2976 (58%)	2342 (58%)	634 (58%)	0.29
**Never smoker**	2194 (42%)	1727 (42%)	467 (42%)	-
**Unknown**	18 (0%)	17 (0%)	1 (0%)	-
**Labs at metastatic diagnosis**				
**CA19-9 (u/mL)**	309.9 (50.7–2769.4)	617 (84–4712)	92.8 (27.8–644.5)	< 0.001
**Albumin (g/dL)**	3.6 (3.2–3.9)	3.6 (3.1–3.9)	3.6 (3.2–4.0)	0.006
**Lymphocytes (× 10**^**9**^**/L)**	1.3 (0.9–1.8)	1.2 (0.9–1.7)	1.3 (0.9–1.9)	0.05
**Neutrophils (× 10**^**9**^**/L)**	4.1 (2.5–6.8)	4.2 (2.5- 7.1)	3.9 (2.5- 6.2)	0.06
**Monocytes (× 10**^**9**^**/L)**	0.6 (0.3- 0.9)	0.6 (0.3–0.9)	0.5 (0.3–0.8)	0.021

### Survival analysis

**Fig. 2:**
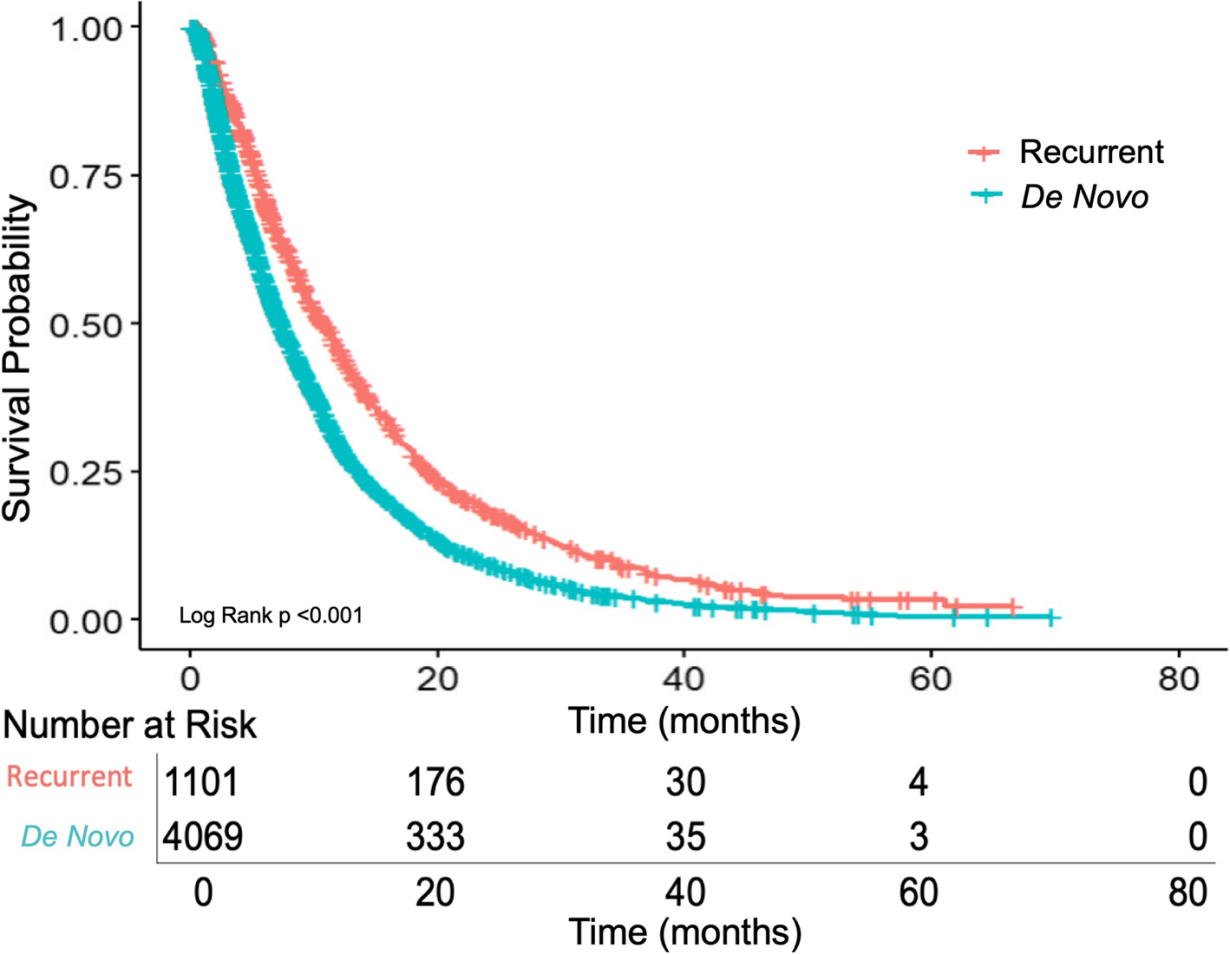
Overall survival from metastatic disease diagnosis of recurrent PDAC versus *de novo* PDAC

**Fig. 3:**
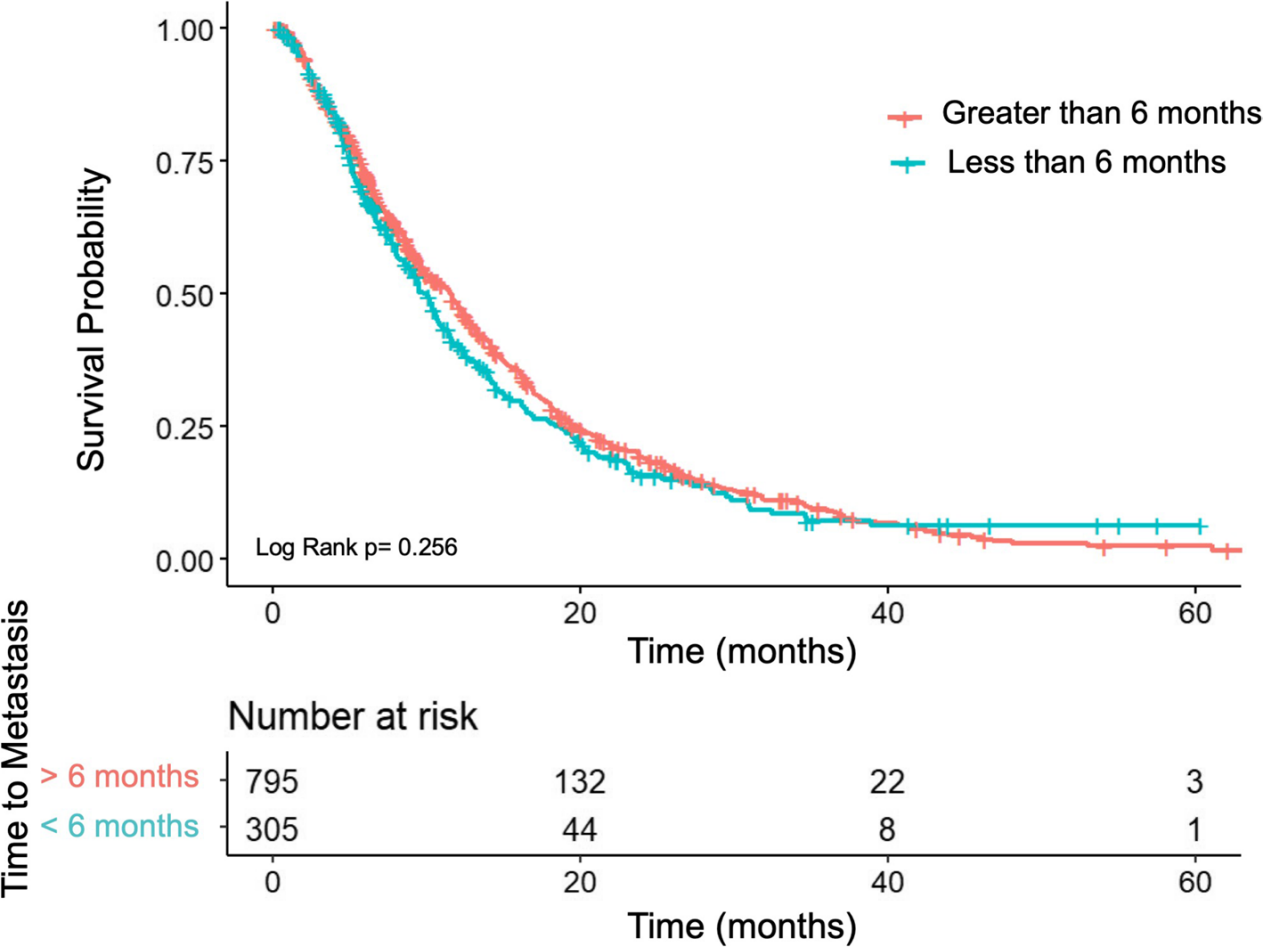
Overall survival from metastatic diagnosis in patients who undergo surgery stratified by time to recurrent disease

Median OS for patients with recurrent PDAC who received systemic therapy was significantly longer than those with *de novo* metastatic PDAC (10.8 months, 95% CI 9.9–11.7 versus 7.3 months, 95% CI 7.0–7.7; *p* < 0.001) (Fig. 2). When controlling for potential confounding variables using a multivariable analysis, there was a significantly greater risk of death in the *de novo* metastatic PDAC group (HR 1.2, 95% CI 1.1–1.3, *p* < 0.001). Key survival outcomes are summarized in Table 2. When comparing survival of patients with recurrent PDAC based on time to development of recurrent disease, no statistically significant difference in OS was identified between those who developed recurrent disease within six months of surgery and those who recurred greater than six months from surgery (10.0 months, 95% CI 8.7 -11.0 versus 11.6 m, 95% CI 10.0–12.5, *p* = 0.26) (Fig. 3).

**Table 2:** Summary of survival outcomes stratified by recurrent versus de novo status

			**Univariable Analysis**			**Multivariable Analysis**		
**PDAC population**	**Overall survival (months)**	**95% CI**	**HR**	**95% CI**	***P*-value**	**HR**	**95% CI**	***P*-value**
Recurrent	10.8	9.9–11.7	Reference			Reference		
*De Novo*	7.3	7.0–7.7	1.48	1.37–1.6	< 0.001	1.2	1.1–1.3	< 0.001

## Discussion

Patients with recurrent metastatic disease represent a significant proportion of the total number of pancreatic cancer patients presenting to oncology clinics. Despite this fact, there is limited data to help guide the management of these patients in the palliative setting. In this study, we examined real-world outcomes of patients with recurrent PDAC based on an analysis of a large US database. As would be expected in a real-world setting, our data demonstrate approximately one-fifth of patients receiving treatment for advanced disease are classified as having recurrent disease following curative-intent resection. When comparing outcomes of patients with recurrent PDAC to those with *de novo* metastatic PDAC, our survival analysis suggests that patients with recurrent PDAC who receive palliative systemic therapy have a significantly greater OS, with a clinically significant absolute difference of approximately three months. Additionally, we did not detect any difference in overall survival in the recurrent patient population based on a recurrence within six months compared to after six months from surgery.

There are multiple potential explanations for the increased overall survival in the recurrent population. It is likely that at least some of the difference in survival between groups in this study is the result of biases inherent to retrospective studies that could not be completely controlled. Specifically, a selection bias may exist in terms of the patients who are selected for surgery. One can hypothesize that patients with better ECOG performance status are selected for surgery, as evidenced by the greater percentage of patients with an ECOG less than 2 in the recurrent group compared to the *de novo* group (65% versus 51%). Additionally, a poor performance status following resection may result in a patient not proceeding with or being offered systemic therapy following a diagnosis of recurrent disease while patients with an equally poor performance status who are treatment naïve may still elect to pursue systemic therapy. It is also possible that our findings are the result of lead-time bias with close surveillance following surgery leading to detection of recurrent disease in patients at an earlier time when they have a smaller overall tumor burden than those diagnosed with *de novo* advanced disease who may have a large intact primary tumor or more extensive metastatic disease. The significant difference in CA 19–9 at time of advanced diagnosis (92.8 U/ml in the recurrent group vs. 617 U/ml in the *de novo* group) supports this hypothesis. Although the data set we utilized did not have data regarding surveillance frequency following surgery, future studies examining the correlation between post-surgical surveillance, CA19-9 levels and overall survival may help guide treatment in a recurrent PDAC population.

Despite these potential biases, it is also important to consider whether our findings could be related to actual differences in the underlying biology of disease in patients with recurrent versus *de novo* disease. When recurrent PDAC tumor samples were sequenced, the genomic landscape shows a heterogeneity of driver mutations between the primary tumor sample and metastasis. Namely, in patients who have undergone adjuvant treatment, samples from their metastatic sites showed an increased tumor mutational burden, as well as enrichment in genetic aberrations in the MAPK or PI3K-AKT pathways. This is in direct contrast to *de novo* stage IV PDAC tumors which showed a homogenous driver mutation pattern between primary and metastatic samples consistent with a clonal population [13]. Peri-operative chemotherapy likely plays a large role in selecting for a certain subpopulation of cells, however tumor microenvironment, *SMAD4*status and immunophenotypes have all been explored as factors contributing to disease recurrence and overall outcome in initially resectable disease [21–23]. This may suggest that patients with recurrent stage IV PDAC should perhaps be thought of differently than *de novo* patients when choosing treatment.

Regardless of the explanation for differences in survival discussed above, what seems clear from our data is that patients with recurrent PDAC appear to do at least as well or better than patients with *de novo* advanced PDAC, even if their disease recurs early after surgery. Thus, we argue that there is little justification for the exclusion of patients with recurrent PDAC in future clinical trials evaluating palliative systemic therapy.

Increasing enrollment in these trials is imperative to improving the outcomes of patients with this disease. While the percentage of patients enrolled in clinical trials for pancreatic cancer has been increasing modestly over the past decade, up from 3.85% in 2011 to 4.15% in 2014, there is still a large imbalance between patient need and number being enrolled [24]. This imbalance is unlikely to be due to difficulty enrolling patients who qualify for trials. A single-center retrospective study in 2014 found that 71% of patients eligible for clinical trials were enrolled [25]. More likely, the low number of patients being enrolled is due to overly restrictive inclusion and exclusion criteria, such as limits on prior therapy or surgery. Indeed, of the patients that called the Pancreatic Cancer Action Network with interest in enrolling in a clinical trial, two-thirds were ineligible due to prior therapy [24, 26]. In the context of a disease where patients can decline rapidly in the absence of systemic therapy, it would be reasonable to allow patients who have received one or two doses of chemotherapy to still be enrolled in first line trials.

From 2011 to 2014 there was an increase in clinical trials for refractory and previously treated disease to 38%. This had the effect of decreasing the time it would take for trials studying this population to complete enrollment from 7.1 years to 6.0 years [24]. However, this is still extremely slow for a disease in which survival is generally on the order of months. Our work shows no difference in overall survival based on when patients recurred after surgery. Future trials evaluating palliative therapies in the advanced disease setting should consider inclusion of patients with recurrent PDAC, even if their recurrence occurs early after surgery. Given potential differences in survival between these populations, trials should stratify any randomization by disease presentation.

This study was based on a well-validated, nationwide real-world database; however, several limitations should be noted. Despite attempts to reduce confounding utilizing a multivariable analysis, it is possible that residual confounding may have contributed to our results. Most notably, data regarding receipt of neoadjuvant or adjuvant treatment was not available for inclusion in our statistical models. Additionally, some incomplete data was present for several variables included in our analysis, such as ECOG status. It should also be noted that in order to compare overall survival between our *de novo* metastatic population and recurrent metastatic population we chose to define overall survival as time from metastatic diagnosis to death. Despite these limitations, we believe this study provides valuable information that can be used to guide discussion of treatment options with patient in the clinic and to help inform the development of more inclusive clinical trials in this devastating disease.

## Conclusions

In conclusion, our data imply that patients with recurrent metastatic PDAC have improved overall survival compared to their *de novo* counterparts. This is regardless of the time to recurrence. As such, one way to improve enrollment in PDAC clinical trials investigating systemic therapies in the palliative setting may be to allow inclusion of patients who relapse within 6 months of resection as our data shows these patients don’t fare worse compared to those who relapse after 6 months.

## Data Availability

The data that support the findings of this study have been originated by Flatiron Health, Inc. These de-identified data may be made available upon request, and are subject to a license agreement with Flatiron Health; interested researchers should contact < DataAccess@flatiron.com > to determine licensing terms.

## Abbreviations

PDAC: Pancreatic ductal adenocarcinoma
OS: Overall survival
PanIN: Pancreatic intraepitheliel neoplasia
